# Anion‐Exchange Membrane Chromatography for the Separation of Empty and Full Adeno‐Associated Viral Capsids

**DOI:** 10.1002/biot.70295

**Published:** 2026-08-17

**Authors:** Luca Ossi, Samuele Delfino, Helena Marie, Michael Schulte, Mattia Sponchioni

**Affiliations:** ^1^ Department of Chemistry Materials and Chemical Engineering Politecnico Di Milano Milano Italy; ^2^ Merck Life Science KGaA Darmstad Germany

**Keywords:** adeno‐associated viruses, anion exchange, design of experiments, elution chromatography, empty/full separation, membrane chromatography, process optimization

## Abstract

The separation of empty and full adeno‐associated viruses (AAVs) is particularly critical because of the similarity between the two. Anion‐exchange chromatography represents a scalable tool, leveraging their slight difference in isoelectric point. However, traditional resin chromatography can only deliver poor resolution, mainly due to the restricted accessibility of the capisds to the bead pores. To cover this gap, in this work, we designed and optimized the separation of empty and full AAVs using anion‐exchange membrane chromatography. The role played by flowrate, gradient slope and wash step parameters on the chromatographic resolution was first investigated with two consecutive designs of experiments (DoE). The response surfaces indicated that high process flowrates, that is 15 membrane volumes (MVs)/min, with a gradient from 115 mM to 250 mM sodium acetate in 350 MVs maximize the chromatographic resolution. Then, preparative experiments showed that fast loading is possible, with residence time as short as 24 s, without product breakthrough. Moreover, membrane loading up to 1.2E17 viral particles (vp)/L_membrane_ could be reached, providing one order of magnitude higher productivity with respect to standard column operations. With these results, we demonstrated the great potential of anion‐exchange membrane chromatography in the polishing of AAVs, overcoming traditional limitations of packed‐bed operations.

## Graphical Abstract and Lay Summary

The anion‐exchange membrane elution chromatography for the separation of empty and full AAVs is explored and optimized. The role played by important parameters like flowrate, gradient slope, membrane loading, and feed residence time is studied in detail. Compared to an equivalent resin separation, the optimized membrane process allowed higher flowrates and resolution, overall contributing to 100× productivity.

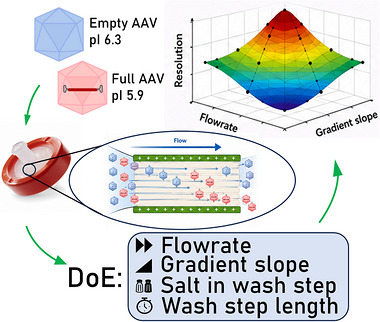

## 1 Introduction

Gene therapies emerged, in the last decades, as fascinating opportunities to treat diseases by introducing specific exogenous genetic sequences into a patient's cell [1]. Such sequences can play different functions, from the replacement of faulty genes with functional ones, the introduction of sequences originally absent in the patient's DNA, to even the silencing of improper ones [2, 3, 4]. To unlock the potential of gene therapies, selective vectors are needed to carry the intact transgene to the involved tissues, protecting it from endonuclease degradation, and to favor its internalization into the patient's cells. Adeno‐associated viruses (AAVs) emerged since the 1980s as particularly suitable vectors, showing specific tissue tropism based on the capsid serotype, low immunogenicity, and low genomic integration into the patient's own DNA and overall high intrinsic safety [5]. AAVs are small (about 25 nm in diameter), non‐enveloped viruses, with a proteinaceous capsid composed by three proteins, namely V1, V2, and V3 in the ratio 1:1:10 [6, 7]. Its core is able to host a transgene of about 4.7 kb in size [8], flanked by Inverted Terminal Repeats (ITRs), forming a T‐shaped hairpin structure acting as the origin of the replication and primers for DNA synthesis [9].

In 1996, the first AAV clinical trial, to treat cystic fibrosis, was undertaken, to which other early‐stage trials focused on monogenic disorders (early 2000s) followed. AAV‐based therapeutics, such as *Glybera* (targeting lipoprotein lipase deficiency), *Luxturna* (targeting Leber congenital Amaurosis type 2) and *Zolgensma* (targeting spinal muscular atrophy) showed much clinical success, receiving first regulatory approval in 2012, 2017, and 2019, respectively. During the early 2020s, other therapies found FDA approval, namely *Roctavian* and *Hemgenix*, targeting hemophilia A and B [10, 11, 12, 13, 14, 15].

A wider spreading of such technology is though fundamentally hindered by production challenges, leading to high therapeutic costs [16, 17, 18, 19]. As a matter of fact, one single treatment can cost up to over one million dollars. AAVs are generally produced via triple transfection of human embryonic kidney cells (HEK293) [20, 21, 22, 23]. By means of a transfection agent, a gene of interest plasmid (pGOI), a RepCap plasmid and a helper plasmid are simultaneously introduced in the production cells. The host cell is then responsible for the capsid assembly and the viral production. Unfortunately, the incorporation of the GOI within the protein capsid is associated to a low success rate, leading to <60% of transgene‐filled capsids (full AAVs) [24, 25]. The main product‐related impurities are, hence, empty capsids, even though other species like partially filled and overfilled capsids may be present in the mixture. The therapeutic effect of empty viruses is up to now not fully understood, some researchers claiming a positive “decoy” effect for the immune system, protecting the transgene‐containing AAVs [26], other advocating a reduced transducing efficiency due to the competition with full AAVs for the cell membrane receptors, associated to a loss of potency of the therapeutic material [27, 28]. Regardless, for a precise dosage of the AAV‐based therapy and to limit the overall viral concentration in an injection, an efficient polishing step is crucial, to enrich the content in full capsids to >70% [29].

Initially, ultracentrifugation in density gradients has been used to carry out this separation [30, 31, 32]. However, this poses major scalability concerns. Hence, chromatographic methods are becoming the preferred solution, counting on well‐assessed methods, available infrastructure and scalable operations [33, 34, 35]. Anion‐exchange chromatography (AEX) is widely recognized as an efficient technology for this separation, leveraging the difference in isoelectric point between empty (p*I* = 6.3) and full capsids (p*I* = 5.9) and being associated to continuous improvements in chromatographic media and modalities [36]. Packed‐bed column chromatography is certainly the most developed and investigated modality, already finding numerous applications in bioprocessing, and reported for the empty/full separation both in the form of single‐column operation [29, 37, 38] and multicolumn countercurrent processes [39, 40, 41]. However, the large size of AAVs (about 25 nm [42]) hinders the achievement of high chromatographic resolution, mainly due to the important pore diffusion limitations in the resin beads, resulting in sub‐optimal particle exploitation and broad peaks [43]. This aspect, combined with the very similar surface charge between empty and full AAVs [44] often leads to an extended co‐elution of the two entities, thus requiring a fine balance between purity and recovery [40, 41]. Internal mass transport limitations can be reduced by using convective flow media. Among them, Dickerson et al. investigated the linear salt gradient elution of AAVs serotype 2 using a monolithic anion exchanger, achieving a four‐fold enrichment in full particles at 90% recovery, a remarkable result supporting the use of these convective media [45]. The anion‐exchange monolith chromatography was further expanded to serotypes 5, 6, 8, and 9, showing promising results in both linear and step‐gradient elutions [46, 47, 48]. An important alternative to monoliths is offered by membrane adsorbents [49, 50]. In such materials, the faster access to the binding sites due to the dominant convective transport allows for sharper peaks and shorter processing times. Indeed, with respect to standard packed‐bed column operations, membrane chromatography supports flowrates that can be up to 10 times higher, thanks to the intrinsic lower pressure drops guaranteed by this system [37, 51, 52].

In this work, we demonstrate the advantages of membrane chromatography in the separation of full from empty AAV2 (polishing). Other works in the literature focused on step‐gradient elutions [53, 54, 55] from a strong anion‐exchange membrane. Despite appealing from a scalability point of view, the proposed processes are typically associated to poor enrichment. Indeed, the very similar physicochemical properties of empty and full AAVs makes the selection of duration and conductivity of the different steps challenging and, even in the best‐case scenarios, full AAV vectors can never be isolated alone. To overcome these challenges, herein we designed and optimized a linear salt gradient elution from a strong anion‐exchange membrane, delivering high full capsid purity. The effect of important process parameters (i.e., flowrate, gradient length, salt concentration in the wash step and its duration) in defining the chromatographic resolution was thoroughly investigated by means of two consecutive designs of experiments (DoE). The screening was carried out in small scale within a high‐performance liquid chromatography (HPLC) set‐up, and the response surfaces obtained from this analysis allowed to define the combination of parameters leading to the maximum chromatographic resolution. The best performing conditions were then validated in a preparative chromatography system, where the additional roles of AAV loading concentration and residence time were evaluated. Finally, a comparison between membrane and packed‐bed chromatography was performed in terms of resolution and productivity at a given full capsid purity. Overall, with this study we aim at demonstrating the great potential of membrane anion‐exchange chromatography to boost the purification capacity of large particles, especially important for those applications in which the cost of the raw materials can seriously mine the economical sustainability of the process.

## 2 Materials and Methods

### 2.1 Materials

Tris(hydroxymethyl)aminomethane (Tris base, purity ≥ 99.8%, MW = 121.14 g/mol), anhydrous magnesium chloride (MgCl_2_, purity ≥ 98.0%, MW = 95.21 g/mol), tetramethylammonium chloride (TMAC, purity ≥ 98.0%, MW = 109.60 g/mol), sodium acetate (NaOAc, purity ≥ 99.0%, MW = 82.03 g/mol), hydrochloric acid (HCl, purity 36.5 ÷ 38.0%), poloxamer 188 and HPLC‐grade water were purchased from Sigma Aldrich.

The mixture of empty/full AAV2 from the capture step to remove process‐related impurities was purchased from Revvity GmbH Germany. The material was produced via triple transfection of HEK293 suspension cell line using lipid‐polymer transfection reagent for efficient incorporation of plasmids within the cells. The gene of interest plasmid (pGOI) contains the coding sequence for green fluorescent protein (GFP) and the human cytomegalovirus (CMV) promoter. Transfection was allowed for 72 h. Cell lysis was performed chemically with AAV‐MAX lysis buffer, to which followed endonuclease treatment. A primary affinity capture was performed with POROS CaptureSelect AAVX by Thermo Fisher. The final product was, then, formulated in phosphate‐buffer saline (PBS) with the addition of 0.014% Tween 20 and sterile filtrated with Acrodisc PES 0.2 µm filter from Cytiva. Two milliliter‐aliquots of the final product with concentration of 1.5E13 capsid particles (cp)/mL were stored at −80°C until use.

### 2.2 AAV Analytics

Empty and full capsid concentrations in the crude material as well as in all fractions collected during linear gradient elutions were analyzed by HPLC, on an Agilent 1200 set‐up. Detection was performed with a UV diode array detector at both 256 and 280 nm and a fluorescence detector (FLD, Agilent 1260 Infinity), with excitation at 280 nm and emission at 350 nm. The analyses were carried out at 30°C with an Agilent Bio SAX NP5 column (4.6 × 50 mm, 5 µm particle size, nonporous particles) connected in series to a Tosoh bioscience TSKgel Q‐STAT SAX column (4.6 × 100 mm, 7 µm particle size, nonporous particles). The HPLC running buffer (Buffer A HPLC) consists of 2 mM MgCl_2_ and 70 mM Tris, whereas the elution buffer (Buffer B HPLC) is obtained from Buffer A HPLC with the addition of 800 mM of TMAC. Both mobile phases were buffered at pH 9.00 ± 0.05 by drop‐wise addition of 5 M HCl and filtered through 0.2 µm polyvinylidene fluoride membranes before usage. Separation conditions consist in a linear gradient elution where Buffer B HPLC is linearly increased from 5% to 35% within 25 min, to which follows a 10 min strip where Buffer B HPLC is held constant at 90% and finally a re‐equilibration step at 5% Buffer B HPLC for additional 10 min. The separation is carried out at a flowrate of 0.7 mL/min and 30°C, with a constant sample volume of 100 µL injected though an autosampler equipped with a 100 µL injection loop. Full and empty AAV concentrations were obtained by integration of the fluorescence detector peak and compared to the area of an injected standard. Full purity is assessed by the ratio of the full AAV area to the overall area.

### 2.3 Membrane Characterization

The chromatographic behavior of the membrane was characterized by evaluating the height equivalent to a theoretical plate (HETP, mm) at significant flowrates, subsequently used for the evaluation of the Van Deemter plot. Pulse injection experiments were performed on an Agilent 1200 HPLC system, using the same detection system described in the previous Section. Flowrates of 0.5, 1.0, 2.5, 5, 10.0, and 15 MVs/min were adopted. Pulse injections (5 µL) of concentrated crude (titer 1.5E13 cp/mL) were performed, with the membrane connected to the HPLC, in non‐binding conditions, achieved with a 450 mM sodium acetate solution at pH = 9.0. From the FLD trace, the statistical moments of order 1 (μ_1_) and of order 2 (*σ* ^2^) of the setup (membrane + system) were evaluated. The same procedure was then applied to the system with the membrane replaced by a zero‐dead‐volume connector, in order to estimate the contribution of capillaries, valves and flow‐cells and hence isolate by difference the role of the membrane, in agreement with Ref. [56].

The detailed calculations are reported in Equations (S1)–(S6).

### 2.4 Design of Experiments (DoE) for Membrane Chromatography Optimization

Membrane chromatography experiments were carried out with a Natrix Q micro (0.2 mL membrane volume (MV), 0.5 mm nominal bed thickness) strong anion‐exchange membrane from Merck Life Science. To design and optimize the separation with a reduced number of experiments, and to consider the interaction between process variables, two consecutive two‐variable circumscribed central‐composite design of experiments (DoE) were performed with the investigation of the following variables: flowrate versus gradient length (DoE1) and percentage of modifier in the wash phase versus its length (DoE2). In both cases, chromatographic resolution was considered as performance separation metric. The experiments for the DoEs were performed through pulse injections (5 µL) of the empty/full AAV mixture (titer 1.5E13 cp/mL), corresponding to 3.75E12 cp/mL_membrane_, in the same HPLC set‐up described in Section 2.2, with the membrane replacing the analytical columns. The running buffer (Buffer A PREP) for these experiments comprised 2 mM MgCl_2_, 70 mM Tris and 0.1%w/v poloxamer 188, while the elution buffer (Buffer B PREP) was the same as Buffer A PREP but with the addition of 500 mM of NaOAc. Both buffers were conditioned to pH 9.00 ± 0.05 by drop‐wise addition of 5 M HCl and filtered through 0.2 µm polyvinylidene fluoride membranes before usage. From the recorded FLD trace, the chromatographic resolution (R_S_) was measured according to Ref. [57] (Equation S7).

Starting from a central experiment, a factorial design defined the variable levels as +1 and −1 of the central point, whereas in the star design the levels were defined as the square root of the number of variables (two in this case). By doing so, a DoE with equidistant points from the design center was generated, as reported in Table 1. The highest and lowest flowrates generated by this design were readjusted to 15.0 and 2.0 MVs/min to test the maximum sustainable flowrate for this membrane and a typical value used in the case of packed‐bed chromatography [40, 41], respectively.

**TABLE 1:** DoE design and validation runs with the values of the investigated variables.

			DoE 1		DoE 2	
ID	Variable 1 (*X*_1_)	Variable 2 (*X*_2_)	Flowrate (MVs/min)	Gradient length (MVs)	B in wash (%)	Wash length (MVs)
1 (center)	0	0	8.5	200	19.0	27.5
2	−1	−1	5.0	100	16.2	11.6
3	+1	−1	11.5	100	21.8	11.6
4	+1	+1	11.5	300	21.8	43.4
5	−1	+1	5.0	300	16.2	43.4
6	0	+√2	8.5	350	19.0	50.0
7	+√2	0	15.0^a^	200	23.0	27.5
8	0	−√2	8.5	50	19.0	5.0
9	−√2	0	2.0^a^	200	15.0	27.5
Validation α			15.0	350	15.0	50.0
Validation β			—	—	23.0	5.0

Abbreviation: MVs, membrane volumes.

^a^ Adjusted values.

For DoE1, no wash phase was performed and the gradient was run from 15.0% to 50.0% Buffer B PREP. For DoE2, the flowrate and gradient slope were fixed to the optimal values obtained from DoE1, specifically to 15.0 MVs/min and 0.1%B/MV. With this constant slope, the gradient was run from the Buffer B PREP used in the wash phase and generated by the design, up to 50.0%.

The response surface of each DoE was evaluated in MATLAB using a quadratic fitting in order to obtain a qualitative response trend.

### 2.5 Preparative Chromatography

Preparative empty/full AAV separations were performed on a ContiChrom CUBE 30+ (YMC ChromaCon). This device is equipped with a conductivity sensor as well as a UV detector measuring the absorbance at 280 nm. Additionally, an external BlueShadow 40D UV/Vis detector (Knauer) was set to record the absorbance at 260 nm at the column outlet. Eluate fractionation was performed by a Foxy R1 fraction collector. Fractionation was performed for all runs both during the linear gradient elution as well as during the loading phase, keeping track of possible breakthrough due to loading speed or feed concentration.

The membrane used for these experiments was the same Natrix Q micro (0.2 mL MV, 0.5 mm nominal bed thickness) reported before. The buffers for all the preparative separations were the same Buffer A PREP and Buffer B PREP described in Section 2.4. The feed material for each chromatographic run was prepared by dilution of the main stock to the desired final concentration in Buffer A PREP, in order to target specific loading concentrations and to lower the solution conductivity to membrane binding conditions. All separation tests were performed by loading 4 mL of diluted feed material. Feed loading flowrate and feed concentration have been individually analyzed, whereas process parameters such as flowrate, gradient slope, percentage of modifier, and length of the wash step were fixed to the optimal values obtained from the DoE analysis (see Section 2.4). The specific conditions for preparative experiments are summarized in Table 2.

**TABLE 2:** Preparative experiments process variables.

Step	Flowrate (MVs/min)	Duration (MVs)	Buffer B Prep (%)
Equilibration	15.0	20.0	23.0
Loading^a^	From 2.5 to 15.0	5.0	0.0
Wash	15.0	5.0	23.0
Elution	15.0	245.0	23.0–42.0
Strip	15.0	45.0	90.0
Re‐equilibration	15.0	30.0	23.0

Abbreviation: MVs, membrane volumes.

^a^ Different feed concentrations were tested from 7.5E11 to 6.0E12 cp/mL to obtain membrane loadings from 1.5E16 to 1.2E17 cp/L_membrane_.

For each run and for each fraction *i*, the full AAV purity (*P*_*i*_, %) was evaluated as the ratio of the full AAV FLD area and the sum of the empty and full AAV FLD areas measured via HPLC analysis. The yield (*Y*_*i*_, %) and the relative process productivity (Prod_*i*_, vg/L_membrane_/h) were calculated as per Equations (1) and (2).

(1) $$Y_{i}\;\left[\%\right]=\frac{C_{i}^{F}V_{i}}{C_{\mathrm{feed}}^{F}V_{\mathrm{feed}}}\cdot 100$$

(2) $$\mathrm{Pro}d_{i}\;\left[\frac{vg}{L_{\mathrm{membrane}}\;h}\right]=\frac{C_{i}^{F}V_{i}}{V_{\mathrm{mem}}t_{\mathrm{proc}}}$$

where $C_{i}^{F}$ and $C_{\mathrm{feed}}^{F}$ (cp/mL) indicate the concentration of full particles in the *i* ^th^ fraction and in the feed, respectively, whereas *V*_*i*_ and *V*_feed_ (mL) are the volumes of the *i* ^th^ fraction and of the loaded feed, respectively. Finally, *V*_mem_ [L_membrane_] is the membrane volume and *t*_proc_ (h) is the total process time. With the unit *vg*, the total number of viral genomes, hence the full AAV particles, are indicated.

A Pareto front purity versus yield is also calculated by pooling together contiguous fractions to simulate different collection windows.

From the moments of the distribution (Equations S1–S3), a gaussian fit was performed on the experimental data (Equations 3 and 4).

(3) $$f_{j}\left(t\right)=\frac{1}{\sqrt{2\pi \sigma_{j}^{2}}}\;e^{-\frac{{\left(t-\mu_{1,j}\right)}^{2}}{2\sigma_{j}^{2}}}\cdot I_{j};\;j=1,\;2$$

(4) $$I_{j}=\sum_{i=1}^{n}C_{j,i}*\frac{V_{i}}{Q}$$

where *f*(*t*) represents the gaussian function, *j* is the index for empty (*j* = 1) or full (*j* = 2) AAVs, *t* is the process time, *C_j_*_,_ *_i_* and *V_i_* are the concentration of AAVs and the volume of the collected fraction *i*, and *Q* is the flowrate, whereas *I_j_* is the total amount of the respective AAVs recovered from the fractionated tests.

The agreement between experimental data and gaussian fit was verified by calculating the coefficient of determination (*R* ^2^).

## 3 Results

### 3.1 Empty/Full AAV Mixture

The AAV2 investigated in this work was produced by triple transfection of suspension HEK293 cells and purified from process‐related impurities by affinity chromatography. The eluate from this capture step is a mixture of empty and full capsids, analyzed via HPLC according to Section 2.2. The FLD chromatogram is reported in Figure S1.

From this chromatogram, the early eluting peak (retention time = 16.6 min) was attributed to empty particles, characterized by a smaller p*I*, while the second peak (retention time = 18.9 min) was attributed to full particles. The resolution achieved via HPLC was *R*_S_ = 0.771.

By integration of the corresponding FLD peaks, the abundance of full capsids was determined as 61%, which motivates the exploration of efficient separation methods to increase its purity in the mixture. Herein, we investigated and optimized the empty/full AAV separation via anion‐exchange membrane chromatography, using a Natrix Q micro adsorbent.

### 3.2 Characterization of the Flow Regime in the Anion‐Exchange Membrane

**FIGURE 1:**
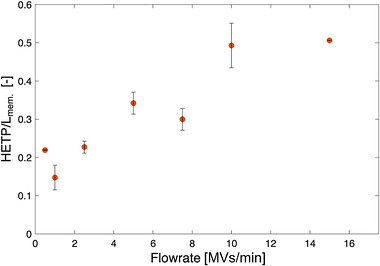
Van Deemter plot of AAV capsids in Natrix Q micro membrane.

To determine the flow regime inside the membrane, the HETP was measured at different flowrates by pulse injections of the AAV empty/full mixture in non‐binding conditions, achieved with 450 mM sodium acetate in the buffer used. The Van Deemter plot reporting the HETP normalized by the nominal thickness of the membrane against the flowrate is reported in Figure 1. It is worth highlighting the high flowrates (i.e., 15.0 MVs/min) that could be achieved, at modest backpressure, with this adsorbent, one order of magnitude higher than those commonly reported for resin‐based separations of the same AAVs [41]. By looking at the plot, despite some experimental variability, it is possible to notice an extended region with constant HETP up to 10.0 MVs/min. This is consistent with the convective flow regime typical of membrane adsorbents, for which the major contribution to band broadening comes from back mixing in the liquid channels. Indeed, constant plate height, although for different molecules, was reported by other authors, in agreement with the results reported herein [58, 59, 60]. On the other side, a decrease in efficiency was recorded at the highest flowrates tested, that is 10.0 and 15.0 MVs/min. This could indicate an increasing contribution of mass transfer limitations, possibly associated to the hampered diffusion of the AAV capsids in the gel phase of the membrane, which is reasonable considering the big size of these particles. Overall, the relatively high absolute value of the HETP is mainly to be associated with the relatively large housing volume of the micro‐sized membrane assembly, that causes important back mixing effects. This fact also contributes to the difficulties in determining the real controlling regime of the membrane, as its own effect could not be decoupled from that of the housing.

### 3.3 Process Optimization Using Two Consecutive DoEs

The membrane chromatography separation of empty and full AAVs was designed and optimized in two steps.

Initially, a DoE (DoE1, see Table 1) considering process flowrate and gradient length as variables (with no wash phase and gradient boundaries fixed between 15% and 50% Buffer B PREP) was performed, to find the combination of these two variables that maximized the chromatographic resolution. The typical FLD chromatogram recorded in these experiments is shown in Figure S2, which was used to evaluate the resolution according to Equation S7. Subsequently, such values have been fixed, and the DoE was repeated exploring the effect of the wash step in the process, namely the percentage of modifier in this process phase, and the length of the step itself. In both cases, the optimal point (or optimal points) was experimentally validated.

**FIGURE 2:**
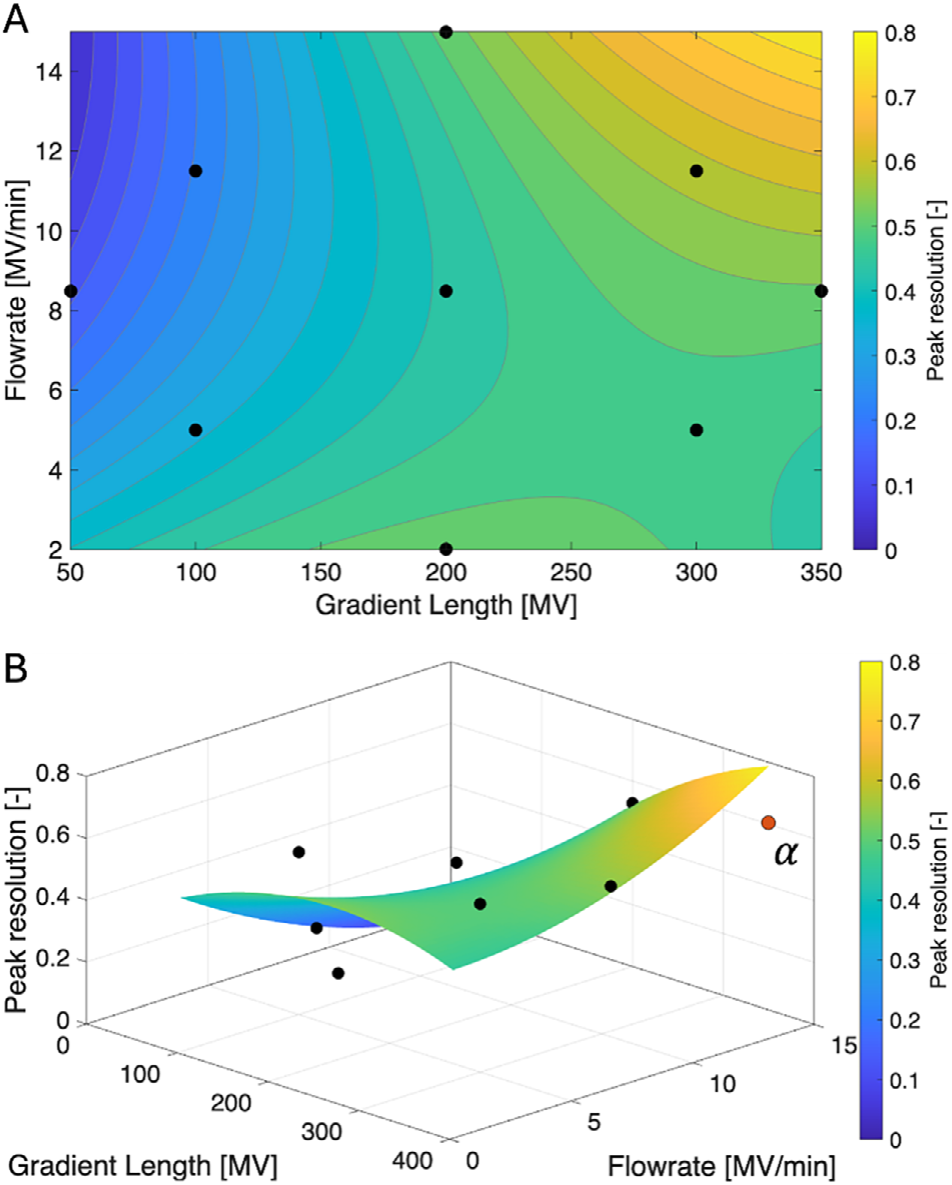
Response surface in terms of peak resolution as a function of flowrate and gradient length. (A) 2D visualization, DoE design points (black dots). (B) 3D visualization, black points indicate the results from the DoE experiments, while the orange dot labelled as 𝛼 indicates result from the validation test performed at 15.0 MVs/min and 350 MV gradient. MV = Membrane volume.

The response surface for the chromatographic resolution as a function of flowrate and gradient duration (DoE1) is shown in Figure 2A. At all the considered flowrates, a monotonic improvement of the separation is obtained by increasing the gradient length, as expected. On the other hand, at fixed gradient length, two different regions can be identified on the plot. Indeed, for short gradients up to 200 MV, the flowrate poorly affects the separation efficiency, as confirmed by regions at equal resolution being almost vertical. On the other hand, for shallower gradients, the resolution significantly increases with the flowrate, showing a point of absolute maximum when both flowrate and gradient length are maximized (within experimental boundaries), that is, 15.0 MVs/min and 350 MV. Additionally, a constant zone is present for intermediate values of gradient lengths (around 250 MV) and flowrate (around 5.0 MVs/min) where these two variables do not have any significant effect on the AAV full and empty separation efficiency. It is worth highlighting that, despite the good separation performance achieved compared to previous works using column chromatography, in no case a baseline separation between empty and full AAVs could be achieved, as testified by a chromatographic resolution < 1.

Figure 2B shows the 3D representation of the response surface, validating the good enough accuracy in the fitting of the experimental results. In addition, this figure reports as orange dot an additional experiment performed to validate the model. This was run at the conditions supposed to maximize the resolution, that is, 350 MV gradient and 15.0 MVs/min. This operation marked a resolution of 0.605, 23.14% lower with respect to the model counterpart but still the highest among the experimental results. This suggests that the general interplay between gradient length and flowrate could be captured by the model.

**FIGURE 3:**
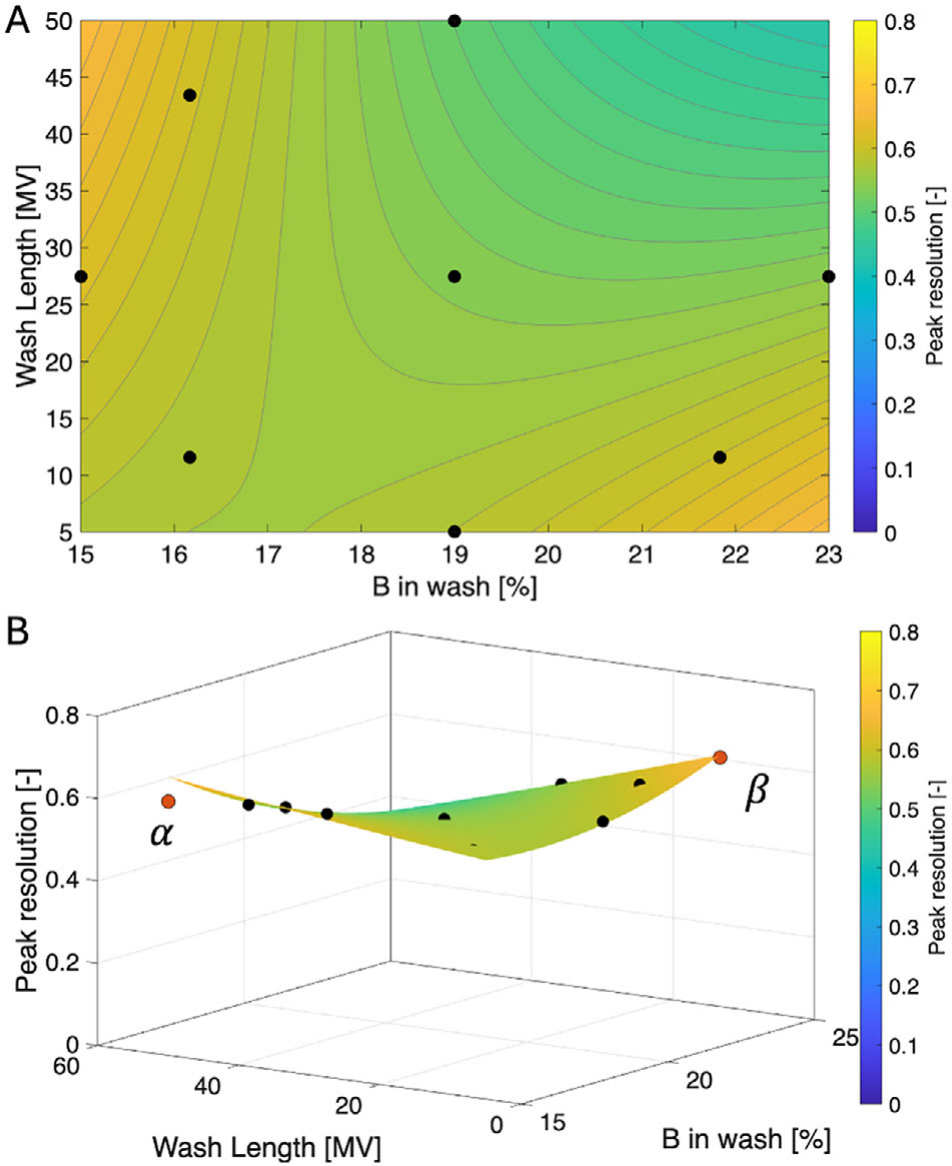
Response surface in terms of peak resolution between wash length and percentage of modifier in the wash step as process variables. (A) 2D visualization, DoE design points (black dots); (B) 3D visualization, black points indicate the experimental results for the DoE tests, the orange dots (𝛼 and 𝛽) indicate the results achieved for the experiments run to validate the optimal process conditions, that is, 𝛼 = 15% B and 50 MV; 𝛽 = 23% B and 5 MV.

Once the optimal combination of gradient duration and flowrate was assessed, these parameters were fixed and a second DoE (DoE2, see Table 1) investigated the interplay between two additional variables, namely the modifier concentration in wash step and wash step length. For this, the response surface is reported in Figure 3. Here, it is worth highlighting that the experimental point at [+1; +1] showed a response value out of range and was hence removed from the surface fitting calculations.

By looking at the response surface, a more complex relationship between the operating variables is obtained in this case. Considering the two diagonals, a saddle point can be spotted, in which one shows a local maximum, and the other a local minimum. Indeed, an increase in wash length has an opposite effect whether it is performed at low or high percentage of modifier, and the same with the variable reversed. Generally speaking, the wash step (isocratic step) shows its highest effectiveness when the modifier concentration is such to guarantee the highest difference in transport speed of the two compounds through the membrane. When its concentration is too low, no movement of the species is induced across the membrane, making this step poorly effective in terms of separation, or in other words, requiring longer duration of this phase to improve the separation. On the other hand, an excessive concentration does lead to fast species transport, but with poor difference between empty and full, reducing only the available membrane volume for the separation (up to the point of elution before gradient starts). In this latter case, the effect leads to the worst separation performance. Based on these considerations, two local maxima for the chromatographic resolution are found corresponding to the highest considered percentage of B (23%) and lowest step length (5 MV) or at lowest considered percentage of B (15%) and highest step length (50 MV). Yet again, a constant zone is present for intermediate wash step lengths and percentages of modifier. Indeed, any increase in wash length performed at 17% of modifier seems not to have any impact on the chromatographic resolution of the peaks, as well as for any increases in modifier content at 15 MV of step length.

The two local maxima found (𝛼 and 𝛽) were experimentally validated and overlayed in Figure 3B with the 3D representation of the response surface. Point 𝛼, corresponding to longer processing time, presents a peak resolution of 0.617, only 8.95% lower than the model prediction. Point 𝛽, corresponding to shorter processing time, presents an even higher peak resolution of 0.665, 1.13% lower with respect to the model prediction.

Based on process productivity considerations, point 𝛽 was chosen as the optimal one and exploited for additional testing on the preparative system, increasing loading concentrations up to two orders of magnitude. As a minor modification to this operational point, since we noticed that the full AAVs completely eluted significantly before the end of the gradient, we stopped the elution at 42% Buffer B instead of 50%, and decreased the gradient duration from 350 MV to 245 MV accordingly to keep the same slope. This duration may still look very high for a potential scale‐up. However, it should be noted that the membrane volume is limited, that is, 0.2 mL in this case, which leads to an elution volume of 50 mL. This is compatible with traditional packed‐bed columns. As a matter of fact, in Ref. [41], exploiting a Fractogel 1 mL prepacked column, the elution volume was 30 mL in the best‐case scenario.

### 3.4 Preparative Elution Experiments to Assess the Influence of Residence Time and Membrane Loading

Based on the results of the process optimization step, the best operating conditions have been translated to the preparative set‐up. Such conditions comprise an equilibration and wash step performed at 23% of Buffer B PREP, a wash step and gradient duration of 5 and 245 MV, respectively, and gradient boundaries 23%– 42%B.

**FIGURE 4:**
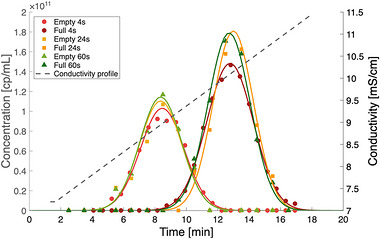
Reconstructed chromatogram including fraction analysis. Dots indicate the experimental AAV concentration (red curves for 4 s residence time, orange curves for 24 s residence time, green curves for 60 s residence time; lighter colors for empty capsids, darker colors for full capsids); solid lines indicate the gaussian fit of species concentration, dashed line indicate the nominal conductivity profile.

Once these process variables were fixed, the impact of loading flowrate on the process performance was investigated first. Three different residence times (RT) were tested, namely 60, 24, and 4 s, by delivering the same amount of mixture that is 4 mL at 7.5E11 cp/mL (total membrane loading of 1.5E16 cp/L_resin_), at progressively increasing flowrates. The following loading times were then used: 20, 2, and 1.33 min from lowest to highest loading flowrate, respectively. On the other hand, the flowrates for all the remaining chromatographic steps were kept constant. The chromatograms achieved after HPLC analysis of the fractions collected during the elution phase are shown in Figure 4.

For the sake of comparison, these chromatograms and the following calculations were obtained by subtracting the loading time from the total run time. For all experimental runs, both for the empty and full AAVs, the first three order statistical moments of the time distribution were calculated. Based on these and according to Equations 3 and 4, the Gaussian fit of the experimental data was generated and its accuracy was evaluated by means of the coefficient of determination *R* ^2^. Figure 4 allows the comparison of the gaussian fit with the experimental results, whereas Table 3 reports the abovementioned parameters as well as the chromatographic resolution calculated from this analysis.

**TABLE 3:** Statistical moments of the distributions, fitting accuracy, and chromatographic resolution during linear gradient elution tests at different loading residence times.

Residence time (s)	Empty AAVs *μ*_1_ (min)	Full AAVs *μ*_1_ (min)	Empty AAVs *σ* ^2^ (min^2^)	Full AAVs *σ* ^2^ (min^2^)	Empty AAVs *R* ^2^ (−)	Full AAVs *R* ^2^ (−)	Peak resolution (−)
60	8.369	12.730	1.761	1.796	0.952	0.991	0.817
24	8.361	12.996	1.802	1.639	0.924	0.989	0.884
4	8.467	12.786	1.926	2.191	0.984	0.997	0.753

Considering that in all cases *R* ^2^ > 0.92 (most of them above 0.98), a Gaussian fit well describes the chromatographic peaks, suggesting an operation in linear isothermal conditions and close to ideal chromatographic behavior.

For each test, four fractions were collected, 1 mL each, toward the end of the loading phase, to assess eventual product breakthrough. No material losses were recorded at any loading residence time, confirming the possibility of keeping a high flowrate also during the loading. Moreover, high full AAV recoveries were measured in all the experiments, 99% in the case of RT 60 s, 96% for RT 24 s, and 93% for RT 4 s, further suggesting that no AAVs were lost during the loading. This is possible due to the low back pressure as well as the convective flow typical of membrane adsorbents, poorly subjected to diffusion limitations. Of significant importance is though the analysis of the elution chromatogram (Figure 4) and the relative peak parameters (Table 3). From the chromatogram, it is immediate to see that for all tested loading residence times, no major changes in the mean elution time (*μ*_1_) was recorded. However, a significant difference was observed in the value of *σ* ^2^. For both empty and full capsids, the peak variance at 4 s RT increased compared to the runs at larger RT. Particularly relevant is the case for the full capsids, whose *σ* ^2^ marked +25% at 4 s RT compared to the case at 24 s. This effect induced also a drop in the peak resolution. Therefore, because of the narrow peak distribution and the relatively high resolution, 24 s was chosen as optimal loading residence time and kept for future tests.

After having defined the optimal residence time, the separation performance was evaluated at different membrane loadings. The same volume (i.e., 4 mL) of empty/full mixture but with different capsid concentrations was fed to the system. The base test was performed with a capsid concentration in the feed of 7.5E11 cp/mL to obtain a membrane loading of 1.5E16 cp/L_membrane_ and is indicated hereinafter as 1×. Additional experiments with capsid suspensions at 3.0E12 and 6.0E12 cp/mL were performed in order to obtain membrane loadings of 6.0E16 and 1.2E17 cp/L_membrane_, which were 4× and 8× with respect to the base case, respectively. For all the experimental runs, fractions were collected during the gradient elution and the empty/full concentration was measured via HPLC. For both empty and full AAV peaks obtained from the fraction analysis, the first three statistical moments were calculated, using Equations S1–S3. Based on these, the gaussian fit of the experimental distributions was obtained through Equations (3) and (4). The quality of the fitting was evaluated in terms of coefficient of determination *R* ^2^. In addition, the peak resolution was calculated and compared across the different experiments in Table 4.

**TABLE 4:** Statistical moments, fitting quality and resolution associated to the separation for tests at different membrane loadings.

Run	Empty AAV *μ*_1_ (min)	Full AAV *μ*_1_ (min)	Empty AAV *σ* ^2^ (min^2^)	Full AAV *σ* ^2^ (min^2^)	Empty AAV *R* ^2^ (−)	Full AAV *R* ^2^ (−)	Peak resolution (−)
1×	16.398	20.556	2.127	2.037	0.985	0.994	0.721
4×	16.711	20.576	2.159	1.744	0.992	0.958	0.693
8×	16.905	20.569	2.918	2.104	0.968	0.983	0.580

**FIGURE 5:**
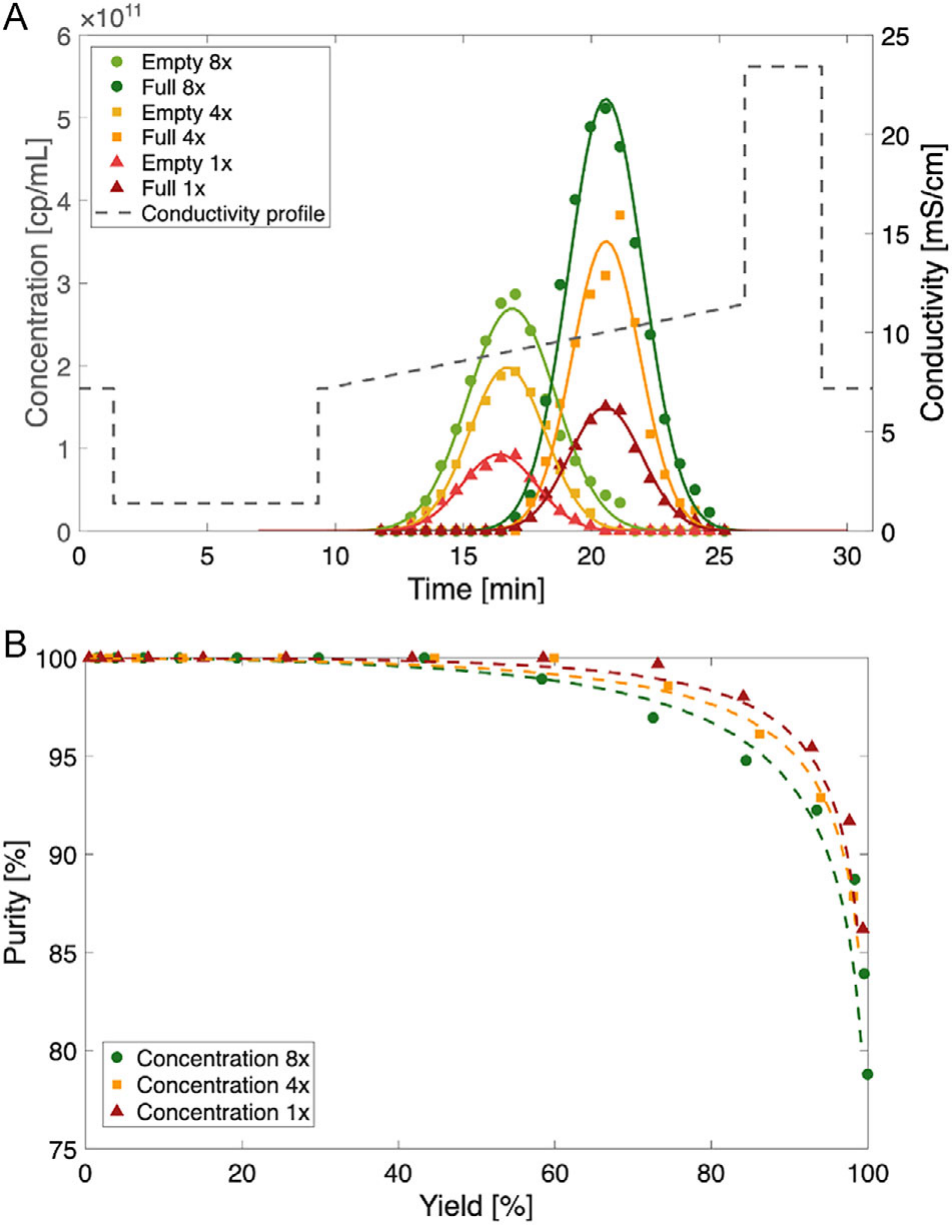
Tests at increasing membrane loadings: (A) Reconstructed chromatograms from fraction analysis. Dots indicate the experimental AAV concentration (red curves for 1×, orange curves for 4×, green curves for 8×; lighter colors for empty capsids, darker colors for full capsids); solid lines indicate the gaussian fit of species concentration, dashed line indicate the nominal conductivity profile. (B) Pareto plots (normalized) between pooled product purity and yield. Dots indicate experimental data; dashed line indicate the model fitting.

Once again, all the gaussian fits showed a *R* ^2^ > 0.95 (most of them above 0.98), stressing again the good data representation and operation in linear isothermal range even at the largest membrane loading. The experimental chromatograms overlayed onto their gaussian fits are represented in Figure 5A.

Regarding the full AAV purification, all three runs yielded a high product recovery. In particular from the lowest to the highest membrane loading conditions, the product recoveries were 96%, 98% and 86%. Additionally, all of the chromatographic runs showed a relatively high resolution between empty and full AAVs, with large portions of the chromatogram where full AAVs eluted with a purity close to 100%. Still, a certain co‐elution between empty and full AAVs was observed regardless of the loading. This partial co‐elution introduced a tradeoff between yield and purity. In fact, the full AAV purity can be maximized by discarding the portion contaminated by empty capsids, penalizing the yield. Alternatively, the recovery can be maximized by incorporating in the product pool also this fraction, at the expenses of a poorer purity. All the intermediate possibilities are reflected in a Pareto front, visible for the different loadings in Figure 5B. This plot simulates the progressive pooling of product fractions, starting from the purest one and moving to the incorporation of less pure fractions. Herein, for a fair comparison, the Pareto front is normalized to achieve 100% yield when all the fractions containing full capsids were aggregated. Figure S3 shows the same plot but with the actual recoveries measured experimentally. From both figures, a more and more severe trade‐off is observed for higher membrane loadings, as a result of the already mentioned more extended overlap between the empty and full capsid peak.

**FIGURE 6:**
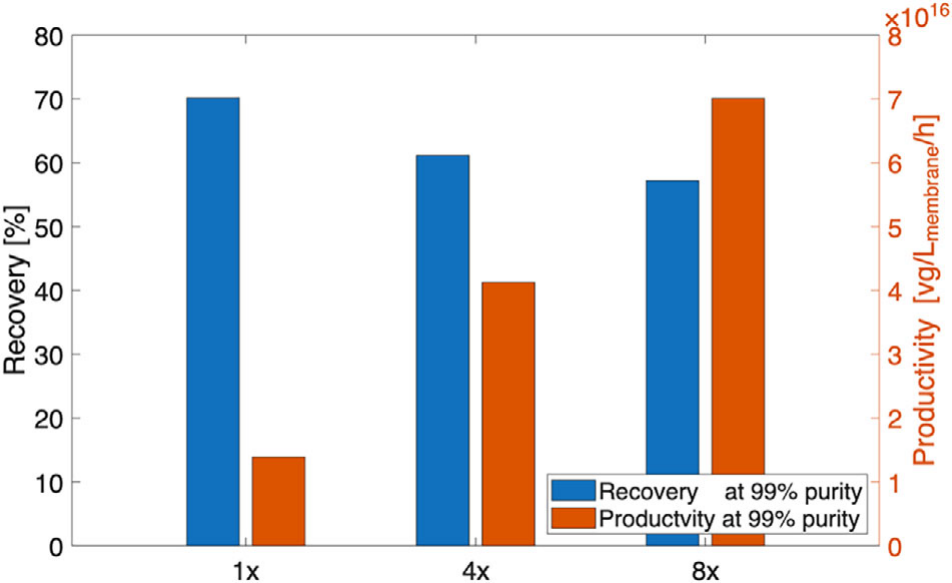
Full AAV recovery at 99% purity in preparative experiments as function of membrane loading (blue bars). Full AAV productivity considering 99% of pooled product purity as function of membrane loading (orange bars).

To better visualize the implications of the different profiles shown in the Pareto plot, process recovery and productivity for a product pool with at least 99% purity are reported together in Figure 6 as a function of membrane loading. The significant decrease in the peak resolution at larger loadings (Table 4) is directly reflected in the recovery at a fixed purity specification. In fact, a monotonic decrease was obtained, from 70% at 1× down to 57% at 8×.

Despite the decrease in product recovery with increased membrane loading, an opposite trend is obtained when considering the full AAV productivity (calculated as in Equation 2) in the same purity conditions. Indeed, from 1.51E16 vp/L_membrane_/h in the base case, an increase to 4.13E16 vp/L_membrane_/h (i.e., +174%) was obtained for the 4× loading titer, up to 7.01E16 vp/L_membrane_/h (i.e., +364%) for the 8× case.

Considering both process aspects presented in Figure 6, the determination of the optimal conditions in this operation cannot be univocally stated. If on the one hand, the increase in loading capsid titer yields an almost linear increase in the productivity, on the other the AAV recovery at a target purity seriously decreases. Considering the high value of such particles and the difficulty in their manufacturing, product recovery may be ultimately prioritized, with the aim of reducing the process costs and pressure on the upstream section. Additionally, it is worth mentioning that by accepting a lower product purity, as visible from the pareto fronts in Figure 5B, both yield and productivity can be significantly enhanced. For example, already a purity specification of 90% allows, for all three loading titers, product recoveries higher than 95%, with productivity increasing linearly up to 1.2E17 vp/L_membrane_/h in the case of the highest concentration (8×).

### 3.5 Comparison of Resin and Membrane Chromatography

**FIGURE 7:**
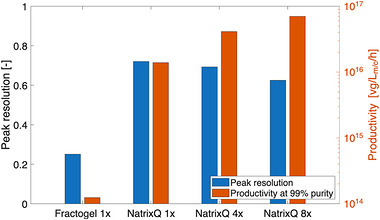
Performance comparison between pre‐packed Fractogel EMD TMAE (S) 1 mL minichrom column and Natrix Q micro 0.2 mL membrane. Blue bars indicate the peak resolution (left axis), orange bars indicate the process productivity considering a purity specification of 99% (right axis).

To further demonstrate the great potential of membrane chromatography in AAV purification, we compared the performance of the optimal process using this modality with that achieved with the strong anion‐exchange resin Fractogel EMD TMAE (S) that we optimized in our previous work [41], operated in 1× loading conditions (4 mL of crude material diluted 1:20, total of 3.0E12 cp injected). The experimental chromatograms for the two operations are shown in Figure S4a. While the column operation allowed to obtain narrower peaks, empty and full capsids extensively co‐eluted in this case. As a result, only <20% of the injected full capsids could be recovered at a purity >99.0%, as visible in the Pareto front reported in Figure S4b. On the other hand, the membrane separation delivered broader peaks. This was attributed to the important back‐mixing in the housing, which is characterized by a relevant volume compared to that of the membrane. This is indeed confirmed by the high value of the HETP in the Van Deemter plot shown in Figure 1. At the same time, the membrane was able to ensure higher resolution between empty and full particles, with recovery of the latter >75% at the imposed purity specification of 99.0%. A side‐by‐side comparison in terms of peak resolution and process productivity (considering a purity specification of 99.0% in full AAV) between column and membrane are visible in Figure 7.

Given the important mass transfer limitations present in the packed‐column chromatography, significant lower processing flowrates are allowed. Indeed, the Fractogel purification was operated at 0.35 mL/min, almost 10 times lower with respect to the flowrate used with the Natrix Q membrane (i.e., 3.0 mL/min). Another remarkable difference lies in the peak resolution, which in the case of the resin was limited to 0.25, while for similar loadings it reached >0.70, as reported in Figure 7 (blue bars). Overall, higher flowrate and better separation were reflected in much higher process productivity when using membranes. When setting a product purity specification of 99.0%, a two‐order‐of‐magnitude increase is obtained by choosing the membrane operation (when compared with same loaded capsids), moving from 1.24E14 vg/L_column_/h up to 1.39E16 vg/L_membrane_/h. Even considering the difference in volume between the two chromatographic systems (1 mL for the column and 0.2 mL for the membrane), still an absolute productivity of 1.24E11 vg/h for the column and a 2.78E12 vg/h for the membrane were recorded, still indicating a difference of a factor 22 between the two chromatographic modalities.

Considering other literature works using anion‐exchange membranes [53, 54, 55], in which the empty/full separation is mainly performed via a two‐step elution protocol, the development of a gradient elution protocol proposed in this work allowed to obtain, at comparable product recoveries (90%), significant increases in purity (product purity > 95%), still maintaining a high process productivity. This could be accessed by a clear understanding of the interplay between different process variables on peak shape and resolution, enabled by the two‐step DoE approach explored in this work.

## 4 Conclusions

In this work, the anion‐exchange membrane separation of empty and full AAVs was designed and optimized on a Natrix Q micro adsorbent. The complex relationship between process variables like elution duration, flowrate, and wash duration were studied by two consecutive DoEs, which allowed with a reasonable number of experiments to develop a model able to accurately predict the influence of these mentioned variables on the chromatographic resolution. The best performing conditions in the two set of investigations indicated/predicted 15.0 MVs/min with elution duration of 350 MVs and 115 mM NaOAc at 5 MVs for the wash step, respectively. Validation tests of these conditions showed good agreement with the predictions obtained by the model. These process conditions were then translated to a preparative set‐up, where the loading conditions, in terms of capsids fed and loading flowrate, were additionally studied. All preparative tests showed symmetric peaks, accurately fitted by a Gaussian distribution (*R* ^2^ > 0.92), suggesting a linear isothermal behavior in conditions close to ideal chromatography.

In all tested loading residence times (60, 24, and 4 s) no capsid losses in the flowthrough were recorded, but in the case of the shortest residence time the peaks showed an increased broadness, leading to a decrease in peak resolution. For this reason, 24 s residence time was chosen as the optimum for the process. Furthermore, membrane loadings of 1.5E16cp/L_membrane_, 6.0E16 cp/L_membrane_, and 1.2E17 cp/L_membrane_ were tested. As result, a monotonic decrease in elution peak resolution was obtained with increasing capsid amounts loaded onto the membrane adsorbent. The process productivity was additionally calculated to evaluate together the optimum obtained by higher capsid loadings, but lower product peak resolution. Indeed, a maximum was obtained for the experiment at 6.0E16 cp/L_membrane_ loading, reaching a productivity of 4.13E16 vg/L_membrane_/h.

The significantly higher processing flowrates allowed by the small mass transport resistances offered by membrane adsorbents, coupled with a mainly convective behavior of such materials, can yield productivity values order of magnitude higher compared to the packed‐bed column counterparts. Such results highlight the great potential that membrane chromatography offers in making AAV processing more efficient and economically sustainable.

## Nomenclature

**Abbreviations**
AAV: Adeno‐associated virus
AEX: Anion‐exchange chromatography
cp: Capsid particles
DoE: Design of experiments
FDA: Food and Drug Administration
FLD: Fluorescence detector
GFP: Green fluorescent protein
HEK293: Human embryonic kidney 293 cells
HETP: Height equivalent to a theoretical plate
HPLC: High‐performance liquid chromatography
ITR: Inverted terminal repeat
MV: Membrane volume
NaOAc: Sodium acetate
PBS: Phosphate buffered saline
PES: Polyether sulfone
Prod: Productivity
pGOI: Plasmid form of the gene of interest
p*I*
: Isoelectric point
*R*_S_
: Chromatographic resolution
RT: Residence time
TMAC: Tetramethyl ammonium chloride
vg: Vector genomes
vp: Viral particles

**Symbols**
*μ*_1_
: First‐order moment of the distribution
*σ* ^2^
: Second‐order moment of the distribution
*P*
: Purity
*R* ^2^
: Coefficient of determination
Y: Yield

## Author Contributions

**Luca Ossi**: validation, investigation, data curation, writing – original draft. **Samuele Delfino**: investigation. **Helena Marie**: writing – review and editing. **Michael Schulte**: resources, supervision, writing – review and editing. **Mattia Sponchioni**: supervision, project administration, writing – review and editing.

## Conflicts of Interest

H. Marie and M. Schulte are affiliated to the company distributing the membrane adsorbent investigated in this work. The authors declare no additional conflict of interests.

## Supporting information



Electronic Supporting Information (ESI) are available at the publisher's website, reporting the chromatographic characterization of the crude AAV mixture, an exemplary chromatogram for the calculation of the resolution from the DoE experiments, the Pareto fronts for the tests at different loadings with the yield not normalized and the comparison of chromatograms and Pareto fronts for column and membrane separations. **Supporting File**: biot70295‐sup‐0001‐SuppMat.docx.

## Data Availability

The data that support the findings of this study are available from the corresponding author upon reasonable request.
