# Supplementary material for: Anion‐Exchange Membrane Chromatography for the Separation of Empty and Full Adeno‐Associated Viral Capsids
Source: Biotechnol J. 2026 Aug 17;21(8):e70295. doi: 10.1002/biot.70295 (PMC13478881; doi:10.1002/biot.70295)
Supplement: Supplementary file 1 [file BIOT-21-e70295-s001.docx]

**Supporting Information for**

**Anion-Exchange Membrane Chromatography for the Separation of Empty and Full Adeno-Associated Viral Capsids**

*Luca Ossi^1^, Samuele Delfino^1^, Helena Marie^2^, Michael Schulte^2^, Mattia Sponchioni^1,*^*

^1^Department of Chemistry, Materials and Chemical Engineering, Politecnico di Milano, Via Bassini 6, Milano, 20133, Italy

^2^Merck Life Science KGaA, Frankfurter Str. 250, Darmstadt, 64293, Germany

^*^Corresponding author: Mattia Sponchioni; Tel.: +39-02-2399-3197; e-mail: [mattia.sponchioni@polimi.it](mailto:mattia.sponchioni@polimi.it)

# Table of Contents

[S1. Column Efficiency	S2](#_Toc233037375)

[S2. Analytical Chromatogram of the Crude Empty/Full Mixture	S3](#_Toc233037376)

[S3. DoE Process Optimization chromatogram	S4](#_Toc233037377)

[S4. Non-normalized Pareto fronts at different loading concentrations	S5](#_Toc233037378)

[S5. Membrane vs. Column chromatography	S6](#_Toc233037379)

# S1. Column Efficiency

From the FLD trace, the first three statistical moments of the distribution were calculated according to **Eqs. 1-3**.

$$\mu_{0}\left[ \frac{cp}{mL}min \right]=\int_{0}^{\infty} C_{FLD}dt$$

(S1)

$$\mu_{1}[min]= \frac{\int_{0}^{\infty} C_{FLD}\cdot t dt}{\mu_{0}}$$

(S2)

$$\sigma^{2}\left[ min^{2} \right]=\frac{\int_{0}^{\infty} C_{FLD}\cdot(t-\mu_{1})^{2} dt}{\mu_{0}}$$

(S3)

Where $C_{FLD}$ is the instantaneous signal measured through the FLD.

Pulse injections experiments were repeated for the system with the membrane connected or replaced by a zero-dead-volume. The contribution of the membrane on $\mu_{1}$and $\sigma^{2}$was calculated as the difference between the corresponding moment of the complete setup and that of the system without membrane, as reported in **Eqs. S4-S5**.

$$\mu_{1,membrane}= \mu_{1,global}- \mu_{1,system}$$

(S4)

$$\sigma_{membrane}^{2}=\sigma_{global}^{2}-\sigma_{system}^{2}$$

(S5)

Finally, the height equivalent to a theoretical plate was calculated from **Eq. S6**.

$$HETP=L_{membrane}\frac{\sigma_{membrane}^{2}}{\mu_{1,membrane}^{2}}$$

(S6)

Where $L_{membrane}$ is the membrane thickness.

# S2. Analytical Chromatogram of the Crude Empty/Full Mixture

Figure S1. Analytical chromatogram of the crude empty/full AAV particles recorded by fluorescence detector with excitation at 280 nm and emission at 350 nm. The separation was performed on an Agilent Bio SAX NP5 column (4.6 x 50 mm, 5 μm nonporous particles) connected to a Tosoh bioscience TSKgel Q-STAT^®^ SAX column (4.6 x 100 mm, 7 μm particle size, nonporous particles).

For this separation, ΔR_t_ = 2.20 min and the resolution was Rs = 0.771.

The quantification method has been taken from literature. (Sommer et al., “Quantification of adeno-associated virus particles and empty capsids by optical density measurement”, Mol. Ther. 7 (2003); Richter et al., “Purity and DNA content of AAV capsids assessed by analytical ultracentrifugation and orthogonal biophysical techniques”, Eur. J. Pharm. Biopharm. 189 (2023)).

# S3. DoE Process Optimization chromatogram

Figure S2. Example chromatogram of an Empty/Full AAV particle separation on a Natrix^®^ Q micro membrane within HPLC set-up, recorded by fluorescence detector with excitation at 280 nm and emission at 350 nm.

Example chromatogram obtained by separation with following conditions: 5 MV wash at 0% B; linear gradient from 0 to 50% B in 200 MV; flowrate 1 mL/min. For this separation, ΔR_t_ = 3.59 min (t_empty_ = 26.65 min, t_full_ = 30.24 min) and baseline width W_empty_ = 8.8 min and W_full_ = 8.2 min. The resolution was evaluated directly on the HPLC software (Agilent Chemstation). The baseline widths are calculated by extending the peak tangents to the baseline (G. Carta et al., “Protein Chromatography: process development and scale-up”, Wiley-VCH; John Wiley, 2010). **Eq. S7** was used for the resolution calculation, which in this case resulted in Rs = 0.422.

$$R_{S}= \frac{{\Delta R}_{t}}{{\frac{1}{2}(W}_{full}+W_{empty})}$$

(S7)

# S4. Non-normalized Pareto fronts at different loading concentrations

Figure S3. Pareto plots between pooled product purity and yield. Dots indicate experimental data; dashed line indicate the model fitting.

The experimental investigation on empty/full AAV separation based on leaded capsid concentration is visible as non-normalized pareto plot in ***Figure S3***. Normalized version is visible in the main text in ***Figure 5b.***

# S5. Membrane vs. Column chromatography

A

B

Figure S4.Comparison between Fractogel^®^ EMD TMAE (S) 1 mL minichrom column and Natrix^®^ Q micro 0.2 mL membrane in terms of: A) chromatogram referred to the separation between empty and full AAVs (blue colors, squares, refer to the column; red color, circles, refer to membrane. Lighter colors refer to empty AAVs, darker to full AAVs). B) Pareto fronts of column (blue, squares) and membrane (red, circles) separations. The dashed line refers to a purity specification of 99.0% considered when evaluating the process performance.
